# Pregnancy-Associated Plasma Protein A (PAPP-A) Concentration in Population of Healthy Young People: Interactions with Tobacco Smoke and Anti-oxidative Status

**DOI:** 10.1007/s12012-018-9479-6

**Published:** 2018-10-09

**Authors:** Magdalena Szumska, Aleksandra Damasiewicz-Bodzek, Justyna Czubilińska, Michał Długaszek, Kaja Gawlik, Anna Krywult, Konrad Synowiec, Tomasz Wielkoszyński, Krystyna Tyrpień-Golder

**Affiliations:** 1 Department of Chemistry, School of Medicine with the Division of Dentistry in Zabrze, Medical University of Silesia, Katowice, Poland; 2 Students Research Group of Chair and Department of Chemistry, Zabrze, Poland; 3 Analytical-Bacteriological Laboratory, NZOZ, Pulmonology Unit, Tarnowskie Góry, Poland

**Keywords:** PAPP-A, Smoking, Medicine students, ELISA

## Abstract

Pregnancy-associated plasma protein A (PAPP-A) is a high-molecular zinc-binding metalloproteinase that was first detected in the serum of pregnant women. It can also be detected in men and non-pregnant women. Recently, a growing interest in determining the concentration of PAPP-A as a marker of oxidative stress and atherosclerotic processes has been observed. Among the factors that could potentially influence the PAPP-A formation is the exposure to tobacco smoke. Some components of tobacco smoke have an immediate effect on the body and also direct influence on the cardiovascular system. The aim of this study was to evaluate the relation between PAPP-A concentration and either passive or active exposure to tobacco smoke in the population of medicine students (*n* = 152). The relation between PAPP-A concentration and chosen markers of inflammatory response and anti-oxidative processes was analyzed. The samples of serum, urine, and saliva were collected and main nicotine metabolites in urine samples were determined using ELISA technique. Comparison of the PAPP-A concentrations in the study group revealed that in the group of active smokers, the concentration of the protein was significantly higher than in the group of passive smokers (*p* = .04) and the group of not-exposed students (*p* = .006). PAPP-A concentration showed significant positive correlation with the values of FRAP and main nicotine metabolites. The evident influence of both active and passive tobacco smoke exposure on PAPP-A levels in the studied population of young people who in general are not included in the group of high-risk cardiovascular incidents, shows how important early prevention of anti-health behaviors is.

## Introduction

The increasing problem of civilization diseases has attracted researchers to focus on finding new markers to predict new emerging health risk issues. Medicine offers wide possibilities in detecting the ongoing process of disease, but still not enough to prevent it. Searching for markers, which could show early changes in physiology of the human body, is now a priority for many scientists in the world.

Pregnancy-associated plasma protein A (PAPP-A) was one of four proteins found at high concentrations in the plasma of pregnant women [1]. Since 1974 its function has been unknown, but it was, and still is, successfully used for screening for fetal trisomy 21 [2]. In the 1990s, researchers found protein with proteolytic activity directed specifically toward insulin-like growth factor (IGF)—binding proteins in culture media from a variety of cells [3–5]. In 1999, Lawrence et al. [6] proved that this protein was PAPP-A. Subsequent studies have demonstrated its important role outside pregnancy.

PAPP-A is a high-molecular-mass (Mr ~ 200,000), zinc-binding matrix metalloproteinase, but the one found in the plasma of pregnant women is different from the one in the plasma of non-pregnant women and men. The first of these circulates in a heterotetrameric form, consisting of two subunits covalently bound to two subunits of the proform of its endogenous inhibitor, the eosinophil major basic protein and is produced by syncytiotrophoblast [7]. Non-pregnant PAPP-A is a homodimeric active form, not bound to the inhibitor [7], and produced by fibroblasts, vascular smooth muscle cells, and activated macrophages [8]. PAPP-A is a member of IGF axis. This axis includes growth hormone (GH), insulin-like growth factors (IGF-1 and -2), their six binding proteins (IGFBP-1-6), and IGFBP proteases. IGFBPs act as IGF carriers and modulate their availability and bioactivity. PAPP-A is IGFBPs’ protease. Its primary substrate is IGFBP-4 [3–5] but it also cleaves IGFBP-2 and IGFBP-5 [9, 10]. This proteolytic activity of PAPP-A is IGF-independent and it cleaves IGFBPs when they are bond to IGFs [6]. This cleaving increases the local levels of free IGFs for subsequent receptor activation and signal transduction [11]. IGFs are involved in the regulation of growth, proliferation, and differentiation of various cell types.

PAPP-A has mainly proved to be an important modulator of local IGFs activity. The other functions of non-pregnant PAPP-A have not been fully known but there is more and more evidence that it plays a role in both physiological and pathological conditions. PAPP-A is potentially proatherosclerotic. It plays an important role in advanced atherosclerosis and is one of the biomarkers of acute coronary syndrome as well as being a highly specific predictor of cardiovascular risk [7, 12–14]. Elevated PAPP-A levels are also associated with diabetes [15, 16], renal diseases [17–21], and cerebrovascular diseases [22]. In 2007, Conover and Bale [23] reported for the first time that PAPP-A was a new anti-aging target. This protein has also been suggested to participate in hormone-related cancer development, such as ovarian, endometrial, and breast tumors [24, 25] and it was reported to be elevated in lung cancer [26]. Several studies have implicated the function of PAPP-A as a key determinant of the cellular proliferation that occurs as response to injury and subsequent inflammatory processes and wound healing [27, 28]. Recently, PAPP-A was taken into consideration as one of the markers of an oxidative stress as yet little is known regarding this [29, 30].

The interest in determining the concentration of PAPP-A as a marker of oxidative stress and atherosclerotic processes results from the fact that its increased concentration has been detected in patients with a history of cardiovascular incidents. Still high mortality rates due to cardiovascular diseases, which have become a noticeable problem in many countries, have drawn the attention of scientists to better understand the pathology of these diseases and search for more specific risk markers. The increasing number of scientific reports has been published in recent years showing the potential usefulness of pregnancy plasma protein A (PAPP-A) as one of these specific markers [12, 13].

It is known that smoking is one of the most important risk factors for cardiovascular diseases [31, 32]. There is still little information on the effects of smoking on the concentration of PAPP-A. It has been proved that smoking during pregnancy decreases PAPP-A [14, 33], but as investigation of the pregnancy-associated plasma protein A concentration in serum of tobacco-smoking men and non-pregnant woman is ambiguous [11, 34, 35], further research is necessary. It has been suggested that, as in the case of pregnant women, in the group of healthy male and non-pregnant women, the exposure to tobacco smoke may also affect PAPP-A concentration [35, 36].

Since the population of young people is particularly exposed to the harmful effects of tobacco smoke constituents, and moreover it has been proven that even a short period of smoking can lead to the endothelium damage, research investigating the effect of tobacco smoke on PAPP-A concentration in serum of young people who do not belong to the group of high cardiovascular risk patients, may constitute an important area for further study [37–39].

The aim of this study was to evaluate the relation between PAPP-A serum concentration and either passive or active exposure to tobacco smoke in the population of young adults as well as the relation between PAPP-A concentration and chosen markers of inflammatory response and anti-oxidative processes.

## Materials and Methods

The study was accepted by the Local Ethics Committee of the Medical University of Silesia (Regulation no. KNW/0022/KB1/183/11). All participants have signed an informed consent.

The study group consisted of 152 students (99 non-pregnant women and 53 men) of the first and the second year of the Faculty of Medicine and Department of Medicine and Dentistry and the second year of Emergency Medicine in Zabrze, Medical University of Silesia in Katowice. Mean age of the study group was 21.1 ± 1.3 years, 21.2 ± 1.4 years for women, and 21.1 ± 1.2 years for men. Blood samples were obtained from elbow veins with disposable vacuum blood collectors (Monovette, Sarstedt). Sera obtained were stored in − 70 °C until analysis. The study also included the collection of urine and saliva samples which were taken (as well as serum samples) during the same sampling day and were stored in − 20 °C until further analysis.

PAPP-A concentrations were measured by the ELISA technique with a commercial DRG’s kit (PAPP-A US (ultra sensitive), EIA-4512, USA). The assay was performed according to the manufacturer’s instructions. The calibration curve was composed of six standards (0–450 ng/ml). The sensitivity of the kit was 0.023 ng/ml.

The concentrations of main nicotine metabolites were determined using the ELISA technique as a screening method which enabled the evaluation of tobacco smoke exposure as well as the division of investigated groups into active and passive smokers. The analysis was performed in urine samples according to the previously described procedure [40].

Based on the test results, the study group was divided into the group of active smokers (the concentration of main metabolites of nicotine > 200 mg/ml of urine), passively exposed to tobacco smoke (the concentration of main metabolites of nicotine from 20 to 200 mg/ml of urine), and the group of unexposed to tobacco smoke (the concentration of main metabolites of nicotine < 20 mg/ml urine).

C-reactive protein (CRP) concentrations analysis in serum samples was performed by the ELISA technique (Human C-Reactive Protein (CRP) ELISA Kit, Cell BioLabs, USA). The ferric reducing/antioxidant power (FRAP) values were measured by the Benzie et al. method [41, 42] in saliva samples.

The results obtained were presented using basic parameters of descriptive statistics. Normal distribution of data was measured using Shapiro–Wilk’s test. Comparisons between two independent groups were done using non-parametric *U* Mann–Whitney test. Independent data between the groups of active smokers, passive smokers, and not-exposed students were compared using analysis of variance or its non-parametric equivalent Kruskal–Wallis test and appropriate post hoc tests. The Spearman’s rank test was used for correlations. The *p* < .05 was considered statistically significant. Calculations were performed with STATISTICA for Windows 10.0 software (StatSoft, Cracow, Poland).

## Results

In the study group of medical students, up to 21.7% of people actively smoked cigarettes, and 42.8% were passively exposed to tobacco smoke. The mean concentrations of main nicotine metabolites determined in the investigated groups are presented in Table 1.

**Table 1:** Main nicotine metabolites in urine of student study groups

Main nicotine metabolites (ng/ml)	Active smokers	Passive smokers	Not-exposed
Median (quartile range)	1039.6 (380.9–2130.1)	29.4 (24.4–42.0)	15.7 (12.6–17.7)
Min–max	219.2–11307.5	21.1–189.9	6.5–20.9

The first step in the analysis of the results was the assessment of possible differences in PAPP-A concentration regarding the gender of investigated group of non-smokers and unexposed to tobacco-smoking students. Comparison of the PAPP-A concentrations between the groups of not-exposed to tobacco smoke women and men showed no statistically significant gender-related differences in the investigated population of young people (medians: 11.7 for women vs. 12.8 for men; *p* = .08). PAPP-A levels also did not differ significantly depending on gender in groups of passive (medians: 12.7 for women vs. 13.1 for men; *p* = .15) and active (medians: 15.1 for women vs. 16.3 for men; *p* = .6) smokers. Therefore, in further data analysis , the division regarding the gender in the individual groups (active, passive, not-exposed students) was not used.

Comparison of the PAPP-A concentrations between the active smokers, passive smokers, and the group of not-exposed to tobacco smoke revealed that in the group of active smokers, the concentration of the protein was significantly higher than in the group of passive smokers (*p* = .04) and the group of not-exposed-to-smoking (*p* = .006). The concentration of PAPP-A was higher in the group of passive smokers compared to the group of not-exposed individuals, however, this difference did not reach statistical significance. The results are shown in Table 2.

**Table 2:** PAPP-A concentration in serum of student study groups

PAPP-A (ng/ml)	Active smokers, *n* = 33 (21.7%)	Passive smokers, *n* = 65 (42.8%)	Not-exposed, *n* = 54 (35.5%)
Median (quartile range)	15.4 (13.5–19.7)*^,#^	12.9 (10.7–15.7)	11.9 (9.9–17.3)
Min–max	9.7–45.9	5.9–45.5	4.65–34.2

*p < .05 active smokers versus passive smokers

^#^*p* < .05 active smokers versus not -exposed

The concentrations of FRAP determined in saliva and serum samples and CRP determined in serum samples did not differ significantly between the groups of active and passive smokers and not-exposed to tobacco smoke students (Table 3).

**Table 3:** FRAP in saliva and CRP in serum of student study groups

Median (quartile range)	Active smokers	Passive smokers	Not-exposed	*p*
FRAP in saliva (µmol/l)	462.0 (408–533)	415.0 (363–484)	444.5 (387–525)	.23
FRAP in serum (µmol/l)	510.5 (446–590)	523.0 (468–560)	503.0 (425–624)	.96
CRP (mg/l)	0.30 (0.28–0.36)	0.36 (0.29–0.72)	0.33 (0.29–0.84)	.14

PAPP-A concentration determined in serum samples showed significant positive correlation with the values of FRAP in saliva samples (but not in serum) and with main nicotine metabolites in urine samples, whereas there was no significant correlation with the concentration of CRP analyzed in serum samples. All these results are summarized in Table 4. The serum CRP values were neither significantly correlated with serum and saliva FRAP values nor with mean concentration of main nicotine metabolites in urine samples. Also, the serum and saliva FRAP concentrations did not significantly correlate with the concentration of mean metabolites of nicotine determined in urine samples.

**Table 4:** Correlation values between PAPP-A and FRAP, main nicotine metabolites, and CRP in biological fluids

	Main nicotine metabolites in urine (ng/ml)	FRAP in serum (µmol/l)	FRAP in saliva (µmol/l)	CRP (mg/l)
PAPP-A in serum (ng/ml)				
*R*	.287	− .037	.185	− .148
*p*	.001	.748	.002	.068

## Discussion

The presence of xenobiotics and their metabolites in the human body is associated with a variety of diseases, including cancers, Parkinson’s and Alzheimer’s disease, diabetes, atherosclerosis, myocardial infarction, as well as in aging. The current study indicates that PAPP-A levels in young adults may be associated with tobacco smoke exposure. Approximately more than 20% of the world population still smokes cigarettes [43]. Tobacco smoking has been considered as the greatest contributor to preventable diseases and premature death, worldwide [44–48]. It is associated with increased incidence of cardiovascular disease (CVD) and is the primary cause of chronic obstructive pulmonary disease [44]. Smoking is also one of the major risk factors for various types of cancer [49, 50]. It is very disturbing that in our study group of young people, the percentage of active smokers is nearly 22%. As future physicians, they should not only communicate information about the health risks of smoking to all their patients but also act as role models by not using tobacco products themselves [51].

The influence of smoking and exposure to tobacco smoke on concentrations of PAPP-A is still not completely understood. So far, the majority of scientific works have focused on the examination of the impact of tobacco exposure on PAPP-A concentration in populations of pregnant smoking and non-smoking women but the results of these studies have been contradictory (reviewed in [52]). The impact of smoking and tobacco smoke exposure on PAPP-A levels outside of pregnancy is still not fully understood.

The present study showed that exposure to tobacco smoke has an influence on PAPP-A concentration in the group of young healthy men and non-pregnant women. The exposure to tobacco smoke either passive or active was evaluated by measuring concentrations of main nicotine metabolites in urine. The cotinine, one of the main nicotine metabolites, level provides a meaningful and quantitative measure of an average human’s exposure to tobacco smoke in active and passive smokers and in non-smokers [53]. Many studies have confirmed the high correlation between cotinine serum and urine concentrations [54]. Correlation between concentrations of PAPP-A and tobacco exposure biomarkers found in this study allows us to conclude that tobacco exposure can affect its level in a significant way. In previous studies, a population consisting of men and women smoking cigarettes revealed that in male subjects, serum PAPP-A levels were significantly lower in smokers than in non-smokers. Similarly, in female subjects, serum PAPP-A levels were lower in smokers than in non-smokers (but the difference was not statistically significant) [35]. Also, in our previous study [55], PAPP-A concentration in serum of smokers (men and women) was lower than in non-smokers group. On the other hand, Joaquin et al. [36] did not observe significant differences in concentrations of PAPP-A between smokers and non-smokers. The discrepancy of results can be explained by the fact that in the study of Coskun et al. [35], Joaquin et al. [36] as well as in our earlier research [55], the division into study groups was based on the data from the interview/survey. In our present work, qualification for individual groups (smokers, passive smokers and not-exposed) was based on the concentration of major metabolites of nicotine in the urine, which seems to better reflect the actual exposure than only survey data.

Smoking also affects systemic inflammation by activating inflammatory cells and increasing levels of circulating inflammatory mediators and pro-inflammatory cytokines [56, 57]. Cigarette smoke has been shown to augment the production of pro-inflammatory tumor necrosis factor α (TNF-α), interleukin-1 (IL-1), -6 (IL-6), and -8 (IL-8) and to decrease the production of anti-inflammatory interleukin-10 (IL-10) [58]. Pro-inflammatory cytokines are potent stimulators for the PAPP-A expression [13]. In cultured human fibroblasts and vascular smooth muscle cells, pro-inflammatory TNF-α and interleukin-1β (IL-1β) induce PAPP-A expression in time- and dose-dependent manner [28, 59]. PAPP-A plays an important role in the development of atherosclerosis. In 2007, Harrington et al. [60] crossed the PAPP-A knockout mice (PAPP-A KO) with apolipoprotein E knockout mice (ApoE KO) (established model for atherosclerosis) and found that KO/KO mice showed a 70–80% reduction in atherosclerotic lesion area compared to ApoE KO mice on a high fat diet. Additionally, the KO/KO mice remain at the initial stage of atherosclerosis, as ApoE KO mice progressively developed into the advanced stage of this process [60]. The trans-genetic overexpression of PAPP-A in vascular smooth muscle cells (VSMCs) of ApoE KO mice directly resulted in accelerated expansion of the atherosclerotic lesion area [61] and PAPP-A KO mice could resist VSMCs proliferation and migration—a sign of advanced atherosclerosis [62]. VSMC is a target cell which secretes PAPP-A under stimulation of pro-inflammatory cytokines [13, 63, 64]. It may explain why tobacco smoke exposure (by stimulation of production and release of pro-inflammatory cytokines) results in an increase of PAPP-A concentrations in studied active smokers population.

In recent years, novel markers of coronary artery disease progression have been confirmed, with circulating levels of PAPP-A standing out as the most prominent indicators of this profile. In 2001, Bayes-Genis et al. [65] first proposed PAPP-A as a candidate biomarker for the acute coronary syndrome. They found that elevated PAPP-A originated from eroded, ruptured, or unstable atherosclerotic plaques. Since then, other studies have confirmed these results [14, 66]. Furthermore, PAPP-A is only minimally expressed in stable plaques [34]. The research cited above has directly confirmed that PAPP-A is synthesized most likely by vascular endothelial and smooth muscle cells. The serum PAPP-A level, as a marker of plaque instability, is a strong independent predictor of cardiovascular events in patients with acute coronary syndrome [12, 14, 34, 67].

The positive correlation of PAPP-A concentration with mean nicotine metabolites concentration as well as the significant increase in the level of this protein in the group of active smokers indicates that PAPP-A should be considered as a marker of future risk of cardiovascular diseases dependent on number of cigarettes smoked, a widely known cause of increased risk of atherosclerosis. The results should be deliberated in planning the assessment of cardiovascular risk using PAPP-A concentration due to the fact that it is significantly higher in sera of active tobacco smokers. Considering PAPP-A as a valuable marker of cardiovascular events risk in case of smoking population, the influence of tobacco smoke exposure should definitely be included in order to avoid unreliable results.

Passive smokers are often not fully aware of the fact that passive exposure to tobacco smoke can also lead to significant increase of the risk of CVD or cancer. The trend towards higher concentration of PAPP-A in the study group of students passively exposed to tobacco smoke cannot exclude the possibility that passive smoking increases the formation and release of this protein. This fact may require further research on a larger sample of the population.

Previous research has shown that PAPP-A concentration in serum samples varies depending on gender (increased in male group) and in the male population it also positively correlates with patients age [35]. This study, however, did not include (when the groups were divided by gender) smoking status. Taking into consideration the fact that male gender is a risk factor for cardiovascular events, the authors cited above [35] have proposed different reference intervals for PAPP-A concentrations in relation to gender (lower for men). In our study group of young adults, the concentration of PAPP-A did not differ in the group of not-exposed in relation to gender, but these values did not significantly differ between the groups of smoking men and women or between groups of passively exposed men and women either. This observation shows that gender is not the factor influencing the concentration of PAPP-A in young adult individuals. Perhaps, the difference in relation to the outcomes of the aforementioned authors [35] results from the fact that the study population of our research was younger and particularly homogeneous regarding the age range.

The impact of exposure to tobacco smoke on C-reactive protein concentrations in serum has also been investigated in recent years. The primary regulators of the pro-inflammatory cytokines release are IL-6, IL-1β, and TNF-α produced by neutrophils and macrophages at the sites of injury [68]. Therefore, CRP could represent the possible link between smoking and the induction of inflammatory pathways [57]. However, the results of studies on the effects of smoking on serum CRP levels are not consistent. Some authors reported a significant increase in the concentration of CRP in smokers compared to non-smokers [69–71], and even a dose-dependent correlation between CRP and smoking habits [72]. However, other studies have not confirmed the existence of such correlations [73] or have only confirmed their existence in men [74]. Similarly, contradictory results were obtained by analyzing the effect of sidestream tobacco smoke on CRP concentration in serum [75–77]. In our study, CRP concentration did not significantly differ between the study groups of active smokers, passive smokers, and unexposed to tobacco smoke. CRP concentration did not significantly correlate to the PAPP-A concentration in serum samples and concentration of main nicotine metabolites in urine samples. These results can only confirm the complexity of cytokine-mediated inflammation.

A test measuring the ferric reducing/antioxidant power, called the FRAP assay, is often used as a method for assessing an antioxidant status of organism [41, 42]. The physiological response to the increased “oxidative stress” is the production and activation of many types of antioxidant agents. Therefore, FRAP is a good indicator of systemic anti-oxidative processes [78]. Studies concerning the evaluation of FRAP concentrations in smokers and non-smokers have not given consistent results either. Some authors have reported a significant increase in FRAP concentrations in smokers [79], others have described the decrease of FRAP level which, however, did not correlate to history of smoking [80]. In our study group, FRAP concentration in serum and saliva did not differ between the groups of active smokers, passive smokers, and students not-exposed to tobacco smoke. Moreover, FRAP concentrations in serum and saliva did not significantly correlate to the CRP values in serum samples nor did it show statistically significant correlation with main nicotine metabolites concentration in urine samples. However, it seems an interesting observation that FRAP concentration in saliva showed positive, significant correlation with the concentration of PAPP-A in serum samples. It can therefore be concluded that the same factors that cause an increase in the concentrations of PAPP-A, including exposure to tobacco smoke components and resulting the increased exposure to free radicals, are also responsible for the escalation of the antioxidant barrier, at least in saliva. This increase in anti-oxidative power certainly expresses the organism’s defense when exposed to oxidative stress. The relatively low strength of this correlation may result from the fact that FRAP values can also be significantly influenced by other factors, such as a diet or vitamins and microelements supplementation.

## Conclusions

To conclude, the value of our work is a large size of the study group and the homogeneity of their age. The classification of these young, healthy individuals into groups of active smokers, passive smokers, and individuals not-exposed to tobacco smoke was made on the basis of the main nicotine metabolites concentration determined in urine samples, which reflects the actual exposure to tobacco smoke. The visible influence of both active and passive tobacco smoke exposure on PAPP-A levels in the investigated population of young people, who in most cases are not included in the group of high cardiovascular incidents risk, shows how important early prevention of anti-health behaviors is, especially for future physicians.

Our study suggests that PAPP-A could be considered as an early prognostic marker of atherosclerosis and cardiovascular diseases. But more advanced conclusions need further investigation. It would be necessary to perform a long-term study concerning occurrence of cardiovascular diseases in patients who had increased PAPP-A concentration at a young age, and investigate its correlation with the beginning of processes leading to atherosclerosis in vessels. More research should be performed regarding the estimation of PAPP-A levels in different populations to define factors influencing this protein concentration in order to create reliable values of intervals on the basis of which the detailed prognosis of health risk could be provided.

Due to the ongoing problem of cardiovascular diseases, scientists are searching not only for most specific markers of CVD, but also for new targets of treatment. PAPP-A seems to fulfill both conditions. Much further research will be required, but in the future, suppression of PAPP-A gene expression could be a way to successfully prevent the development of atherosclerosis, and ultimately reduce mortality from cardiovascular diseases.

## References

[1] Lin T-M, Halbert SP, Spellacy WN (1974). Measurement of pregnancy-associated plasma proteins during human gestation. Journal of Clinical Investigation.

[2] van Heesch PN, Struijk PC, Laudy JA, Steegers EA, Wildschut HI (2010). Estimating the effect of gestational age on test performance of combined first-trimester screening for Down syndrome: A preliminary study. Journal of Perinatal Medicine.

[3] Fowlkes J, Freemark M (1992). Evidence for a novel insulin-like growth factor (IGF)-dependent protease regulating IGF-binding protein-4 in dermal fibroblasts. Endocrinology.

[4] Conover CA, Kiefer MC, Zapf J (1993). Posttranslational regulation of insulin-like growth factor binding protein-4 in normal and transformed human fibroblasts. Insulin-like growth factor dependence and biological studies. Journal of Clinical Investigation.

[5] Durham SK, Kiefer MC, Riggs BL, Conover CA (1994). Regulation of insulin-like growth factor binding protein 4 by a specific insulin-like growth factor binding protein 4 proteinase in normal human osteoblast-like cells: Implications in bone cell physiology. Journal of Bone Mineral Research.

[6] Lawrence JB, Oxvig C, Overgaard MT, Sottrup-Jensen L, Gleich GJ, Hays LG (1999). The insulin-like growth factor (IGF)-dependent IGF binding protein-4 protease secreted by human fibroblasts is pregnancy-associated plasma protein-A. Proceedings of the National Academy of Sciences of the United States of America.

[7] Bayes-Genis A, Conover CA, Schwartz RS (2000). The insulin-like growth factor axis: A review of atherosclerosis and restenosis. Circulation Research.

[8] Biasucci LM, Rizzello V (2006). Pregnancy-associated plasma protein-A: Do specific markers of vascular or plaque activation exist, and do we really need them?. Clinical Chemistry.

[9] Laursen LS, Overgaard MT, Soe R, Boldt HB, Sottrup-Jensen L, Giudice LC (2001). Pregnancy-associated plasma protein-A (PAPP-A) cleaves insulin-like growth factor binding protein (IGFBP)-5 independent of IGF: Implications for the mechanism of IGFBP-4 proteolysis by PAPP-A. FEBS Letter.

[10] Monget P, Mazerbourg S, Delpuech T, Maurel MC, Manière S, Zapf J (2003). Pregnancy-associated plasma protein-A is involved in insulin-like growth factor binding protein-2 (IGFBP-2) proteolytic degradation in bovine and porcine preovulatory follicles: Identification of cleavage site and characterization of IGFBP-2 degradation. Biology of Reproduction.

[11] Boldt HB, Conover CA (2007). Pregnancy-associated plasma protein-A (PAPP-A): A local regulator of IGF bioavailability through cleavage of IGFBPs. Growth Hormone and IGF Research.

[12] Consuegra-Sanchez L, Fredericks S, Kaski JC (2009). Pregnancy-associated plasma protein-A (PAPP-A) and cardiovascular risk. Atherosclerosis.

[13] Li Y, Zhou C, Zhou X, Song L, Hui R (2013). PAPP-A in cardiac and non-cardiac conditions. Clinical Chimica Acta.

[14] Lund J, Qin QP, Ilva T, Petterssson K, Voipio-Pulkki LM, Porela P (2003). Circulating pregnancy-associated plasma protein A predicts outcome in patients with acute coronary syndrome but no troponin I elevation. Circulation.

[15] Aso Y, Okumura K, Wakabayashi S, Takebayashi K, Taki S, Inukai T (2004). Elevated pregnancy-associated plasma protein-a in sera from type 2 diabetic patients with hypercholesterolemia: Associations with carotid atherosclerosis and toe-brachial index. Journal of Clinical Endocrinology and Metabolism.

[16] Stulc, T., Skrha, J., Malbohan, I., Fialová, L., & Ceška, R. (2003) Increased levels of pregnancy-associated plasma protein-A in patients with hypercholesterolemia and diabetes: The effect of lipid lowering. 25th Congress of the European Society of Cardiology, Conference Paper Vol 24, p. 458.

[17] Fialova L, Kalousova M, Soukupova J, Sulkova S, Merta M, Jelinkova E (2004). Relationship of pregnancy-associated plasma protein-A to renal function and dialysis modalities. Kidney Blood Pressure Research.

[18] Kalousova M, Horejsi M, Fialova L, Soukupova J, Sulkova S, Malbohan I (2004). Increased levels of pregnancy-associated plasma protein A are associated with mortality in hemodialysis patients: Preliminary results. Blood Purification.

[19] Coskun A, Bicik Z, Duran S, Alcelik A, Soypacaci Z, Yavuz O (2007). Pregnancy-associated plasma protein A in dialysis patients. Clinical Chemistry Laboratory Medicine.

[20] Coskun A, Duran S, Apaydin S, Bulut I, Sariyar M (2007). Pregnancy-associated plasma protein-A: Evaluation of a new biomarker in renal transplant patients. Transplant Proceedings.

[21] Etter C, Straub Y, Hersberger M, Raz HR, Kistler T, Kiss D (2010). Pregnancy-associated plasma protein-A is an independent short-time predictor of mortality in patients on maintenance haemodialysis. European Heart Journal.

[22] Fialova L, Pileckova N, Bauer J, Soukupová J, Kalousová M, Malbohan I (2006). Pregnancy-associated plasma protein-A in patients with cerebrovascular diseases: A pilot study. Prague Medical Report.

[23] Conover CA, Bale LK (2007). Loss of pregnancy-associated plasma protein A extends lifespan in mice. Aging Cell.

[24] Kalli KR, Chen BK, Bale LK, Germand E, Overgaard MT, Oxvig C (2004). Pregnancy-associated plasma protein-A (PAPP-A) expression and insulin-like growth factor binding protein-4 protease activity in normal and malignant ovarian surface epithelial cells. International Journal of Cancer.

[25] Ryan AJ, Napoletano S, Fitzpatrick PA, Currid CA, O’Sullivan NC, Harmey JH (2009). Expression of a protease-resistant insulin-like growth factor-binding protein-4 inhibits tumour growth in a murine model of breast cancer. British Journal of Cancer.

[26] Bulut I, Coskun A, Ciftci A, Cetinkaya E, Altiay G, Caglar T (2009). Relationship between pregnancy-associated plasma protein-A and lung cancer. American Journal of Medical Sciences.

[27] Chen BK, Leiferman KM, Pittelkow MR, Overgaard MT, Oxvig C, Conover CA (2003). Localization and regulation of pregnancy-associated plasma protein A expression in healing human skin. Journal of Clinical Endocrinology and Metabolism.

[28] Resch ZT, Chen BK, Bale LK, Oxvig C, Overgaard MT, Conover CA (2004). Pregnancy-associated plasma protein a gene expression as a target of inflammatory cytokines. Endocrinology.

[29] Finkel T, Holbrook NJ (2000). Oxidants, oxidative stress and the biology of ageing. Nature.

[30] Resch ZT, Oxvig C, Bale LK, Conover CA (2006). Stress-activated signaling pathways mediate the stimulation of pregnancy-associated plasma protein-A expression in cultured human fibroblasts. Endocrinology.

[31] Alshehri AM, Azoz AM, Shaheen HA, Farrag YA, Khalifa MAA, Youssef A (2013). Acute effects of cigarette smoking on the cardiac diastolic functions. Journal of Saudi Heart Association.

[32] Samet JM (2013). Tobacco smoking: The leading cause of preventable disease worldwide. Thoracic Surgery Clinics.

[33] Chelchowska M, Maciejewski T, Gajewska J, Ambroszkiewicz J, Laskowska-Klita T, Leibschang J (2012). The pregnancy-associated plasma protein A and insulin-like growth factor system in response to cigarette smoking. Journal of Maternal Fetal Neonatal Medicine.

[34] Heeschen C, Dimmeler S, Hamm CW, Fichtlscherer S, Simoons ML, Zeiher AM (2005). Pregnancy-associated plasma protein-A levels in patients with acute coronary syndromes: Comparison with markers of systemic inflammation, platelet activation, and myocardial necrosis. Journal of the American College of Cardiology.

[35] Coskun A, Serteser M, Duran S, Inal TC, Erdogan BE, Ozpinar A (2013). Reference interval of pregnancy-associated plasma protein-A in healthy men and non-pregnant women. Journal of Cardiology.

[36] Joaquin C, Granada ML, Pastor C, Castell C, Puig R, Alonso N (2013). Pregnancy-associated plasma protein-A is related to gender and to adipocytokine levels: Results of the Health Survey of Catalonia. Clinical Endocrinology (Oxford).

[37] Larsman P, Eklof M, Torner M (2012). Adolescents’ risk perceptions in relation to risk behavior with long-term health consequences; antecedents and outcomes: A literature review. Safety Science.

[38] Levit RD, Reynolds HR, Hochman JS (2011). Cardiovascular disease in young women: A population at risk. Cardiology Review.

[39] Mansikkaniemi K, Juonala M, Taimela S, Hirvensalo M, Telama R, Huupponen R (2012). Cross-sectional associations between physical activity and selected coronary heart disease risk factors in young adults. The Cardiovascular Risk in Young Finns Study. Annals of Medicine.

[40] Wielkoszynski T, Tyrpien K, Szumska M (2009). The enzyme-linked immunosorbent assay (ELISA) method for nicotine metabolites determination in biological fluids. Journal of Pharmaceutical Biomedical Analysis.

[41] Benzie IF, Strain JJ (1999). Ferric reducing/antioxidant power assay: Direct measure of total antioxidant activity of biological fluids and modified version for simultaneous measurement of total antioxidant power and ascorbic acid concentration. Methods Enzymology.

[42] Benzie IF, Strain JJ (1996). The ferric reducing ability of plasma (FRAP) as a measure of “antioxidant power”: The FRAP assay. Analytical Biochemistry.

[43] Basu S, Stuckler D, Bitton A, Glantz SA (2011). Projected effects of tobacco smoking on worldwide tuberculosis control: Mathematical modelling analysis. BMJ.

[44] Swan GE, Lessov-Schlaggar CN (2007). The effects of tobacco smoke and nicotine on cognition and the brain. Neuropsychology Review.

[45] Goncalves RB, Coletta RD, Silverio KG, Benevides L, Casati MZ, Sliva JS (2011). Impact of smoking on inflammation: Overview of molecular mechanisms. Inflammation Research.

[46] WHO. *Global report: Mortality attributable to tobacco*. http://whqlibdoc.who.int/publications/2012/9789241564434_eng.pdf.

[47] Jha P, Ramasundarahettige C, Landsman V, Rostron B, Thun M, Anderson RN (2013). 21st-century hazards of smoking and benefits of cessation in the United States. New England Journal of Medicine.

[48] Centers of Disease Control and Prevention (CDC) (2008). Smoking-attributable mortality, years of potential life lost, and productivity losses—United States, 2000–2004. MMWR.

[49] Kushi LH, Byers T, Doyle C, Bandera EV, McCullough M, McTiernan A (2006). American Cancer Society Guidelines on Nutrition and Physical Activity for cancer prevention: Reducing the risk of cancer with healthy food choices and physical activity. CA: Cancer Journal of Clinicians.

[50] Ezzati M, Henley SJ, Lopez AD, Thun MJ (2005). Role of smoking in global and regional cancer epidemiology: Current patterns and data needs. International Journal of Cancer.

[51] Rigotti NA, Clair C (2013). Managing tobacco use: The neglected cardiovascular disease risk factor. European Heart Journal.

[52] Gajewska J, Chelchowska M, Ceran A, Ambroszkiewicz J, Laskowska-Klita T (2010). The influence of tobacco smoking on concentration of the pregnancy-associated plasma protein A (PAPP-A) in pregnant women. Przegland Lekarski.

[53] Villaverde A, Parra V, Estevez M (2014). Oxidative and nitrosative stress induced in myofibrillar proteins by a hydroxyl-radical-generating system: Impact of nitrite and ascorbate. Journal of Agricultural and Food Chemistry.

[54] Benowitz NL (1999). Biomarkers of environmental tobacco smoke exposure. Environmental Health Perspectives.

[55] Szumska M, Tyrpień K, Wielkoszynski T (2013). Evaluation of the influence of exposure to tobacco smoke on the concentration of the pregnancy-associated plasma protein A in the population of healthy men and non-pregnant women. Przegland Lekarski.

[56] Rom O, Avezov K, Aizenbud D (2013). Cigarette smoking and inflammation revisited. Respiratory Physiology and Neurobiology.

[57] Yanbaeva DG, Dentener MA, Creutzberg EC (2007). Systemic effects of smoking. Chest.

[58] Arnson Y, Shoenfeld Y, Amital H (2010). Effects of tobacco smoke on immunity, inflammation and autoimmunity. Journal of Autoimmunity.

[59] Conover CA, Bale LK, Harrington SC (2006). Cytokine stimulation of pregnancy-associated plasma protein A expression in human coronary artery smooth muscle cells: Inhibition by resveratrol. American Journal of Physiology Cell Physiology.

[60] Harrington SC, Simari RD, Conover CA (2007). Genetic deletion of pregnancy-associated plasma protein-A is associated with resistance to atherosclerotic lesion development in apolipoprotein E-deficient mice challenged with a high-fat diet. Circulation Research.

[61] Conover CA, Mason MA, Bale LK (2010). Transgenic overexpression of pregnancy-associated plasma protein-A in murine arterial smooth muscle accelerates atherosclerotic lesion development. American Journal of Physiology Heart and Circulatory Physiology.

[62] Resch ZT, Simari RD, Conover CA (2006). Targeted disruption of the pregnancy-associated plasma protein-A gene is associated with diminished smooth muscle cell response to insulin-like growth factor-I and resistance to neointimal hyperplasia after vascular injury. Endocrinology.

[63] Delafontaine P, Song YH, Li Y (2004). Expression, regulation, and function of IGF-1, IGF-1R, and IGF-1 binding proteins in blood vessels. Arteriosclerosis Thrombosis and Vascular Biology.

[64] Conover CA, Harrington SC, Bale LK (2007). Surface association of pregnancy-associated plasma protein-A accounts for its colocalization with activated macrophages. American Journal of Physiology Heart Circulatory Physiology.

[65] Bayes-Genis A, Conover CA, Overgaard MT (2001). Pregnancy-associated plasma protein A as a marker of acute coronary syndromes. New England Journal of Medicine.

[66] Sangiorgi G, Mauriello A, Bonanno E (2006). Pregnancy-associated plasma protein-a is markedly expressed by monocyte-macrophage cells in vulnerable and ruptured carotid atherosclerotic plaques: A link between inflammation and cerebrovascular events. Journal of the American College of Cardiology.

[67] Bonaca MP, Scirica BM, Sabatine MS (2012). Prospective evaluation of pregnancy-associated plasma protein-a and outcomes in patients with acute coronary syndromes. Journal of the American College of Cardiology.

[68] Tonstad S, Cowan JL (2009). C-reactive protein as a predictor of disease in smokers and former smokers: A review. International Journal of Clinical Practice.

[69] Wannamethee SG, Lowe GD, Shaper AG (2005). Associations between cigarette smoking, pipe/cigar smoking, and smoking cessation, and haemostatic and inflammatory markers for cardiovascular disease. European Heart Journal.

[70] Ohsawa M, Okayama A, Nakamura M (2005). CRP levels are elevated in smokers but unrelated to the number of cigarettes and are decreased by long-term smoking cessation in male smokers. Preventive Medicine.

[71] O’Loughlin J, Lambert M, Karp I (2008). Association between cigarette smoking and C-reactive protein in a representative, population-based sample of adolescents. Nicotine & Tobacco Research.

[72] Lowe GD, Sweetnam PM, Yarnell JW (2004). C-reactive protein, fibrin D-dimer, and risk of ischemic heart disease: The Caerphilly and Speedwell studies. Arteriosclerosis Thrombosis and Vascular Biology.

[73] Helmersson J, Larsson A, Vessby B (2005). Active smoking and a history of smoking are associated with enhanced prostaglandin F(2alpha), interleukin-6 and F2-isoprostane formation in elderly men. Atherosclerosis.

[74] Frohlich M, Sund M, Lowel H (2003). Independent association of various smoking characteristics with markers of systemic inflammation in men. Results from a representative sample of the general population (MONICA Augsburg Survey 1994/95). European Heart Journal.

[75] Wilkinson JD, Lee DJ, Arheart KL (2007). Secondhand smoke exposure and C-reactive protein levels in youth. Nicotine & Tobacco Research.

[76] Panagiotakos DB, Pitsavos C, Chrysohoou C (2004). Effect of exposure to secondhand smoke on markers of inflammation: The ATTICA study. American Journal of Medicine.

[77] Venn A, Britton J (2007). Exposure to secondhand smoke and biomarkers of cardiovascular disease risk in never-smoking adults. Circulation.

[78] Goraca A, Skibska B (2005). Plasma antioxidant status in healthy smoking and non-smoking men. Bratislavske Lekarske Listy.

[79] Miri R, Saadati H, Ardi P (2012). Alterations in oxidative stress biomarkers associated with mild hyperlipidemia and smoking. Food and Chemical Toxicology.

[80] Ranjbar A, Rajabian H, Jand Y (2004). The comparison of oxidative stress between smokers and nonsmokers. Arak Medical University Journal.

